# The effect of simulation-based training on problem-solving skills, critical thinking skills, and self-efficacy among nursing students in Vietnam: a before-and-after study

**DOI:** 10.3352/jeehp.2024.21.24

**Published:** 2024-09-23

**Authors:** Tran Thi Hoang Oanh, Luu Thi Thuy, Ngo Thi Thu Huyen

**Affiliations:** Faculty of Nursing, Da Nang University of Medical Technology and Pharmacy, Da Nang, Vietnam; Hallym University, Korea

**Keywords:** Critical thinking, Nursing education, Problem solving, Self efficacy, Simulation training

## Abstract

**Purpose:**

This study investigated the effect of simulation-based training on nursing students’ problem-solving skills, critical thinking skills, and self-efficacy.

**Methods:**

A single-group pretest and posttest study was conducted among 173 second-year nursing students at a public university in Vietnam from May 2021 to July 2022. Each student participated in the adult nursing preclinical practice course, which utilized a moderate-fidelity simulation teaching approach. Instruments including the Personal Problem-Solving Inventory Scale, Critical Thinking Skills Questionnaire, and General Self-Efficacy Questionnaire were employed to measure participants’ problem-solving skills, critical thinking skills, and self-efficacy. Data were analyzed using descriptive statistics and the paired-sample t-test with the significance level set at P<0.05.

**Results:**

The mean score of the Personal Problem-Solving Inventory posttest (127.24±12.11) was lower than the pretest score (131.42±16.95), suggesting an improvement in the problem-solving skills of the participants (t_172_=2.55, P=0.011). There was no statistically significant difference in critical thinking skills between the pretest and posttest (P=0.854). Self-efficacy among nursing students showed a substantial increase from the pretest (27.91±5.26) to the posttest (28.71±3.81), with t_172_=-2.26 and P=0.025.

**Conclusion:**

The results suggest that simulation-based training can improve problem-solving skills and increase self-efficacy among nursing students. Therefore, the integration of simulation-based training in nursing education is recommended.

## Graphical abstract

[Fig f2-jeehp-21-24]

## Introduction

### Background

Simulation-based training methods have emerged as a pivotal solution to address challenges in nursing education. This teaching approach replicates real-life scenarios, enabling students to apply their knowledge and skills in controlled and supervised settings. Engaging in simulations provides a safe learning environment where students can practice without compromising patient safety. Moreover, this teaching method allows students to receive immediate and constructive feedback from instructors and peers, facilitating continuous improvement of their skills. Widely adopted in nursing education, simulation-based training has been shown to improve the performance of nursing procedures, increase clinical competence, and enhance a range of essential abilities such as problem-solving skills, critical thinking skills, and self-efficacy among students [1,2].

Problem-solving skills involve identifying, assessing, and analyzing a problem to select the best solution to overcome internal and external challenges [3]. It is one of the crucial non-technical skills targeted in nursing education. Simulation-based training offers a valuable platform where students can engage in realistic scenarios, allowing them to practice and improve their problem-solving abilities [2].

In addition, critical thinking skills are fundamental for ensuring safe and effective patient care. Critical thinking involves thoughtfully considering evidence, contexts, concepts, methodologies, and criteria to make informed judgments about objects or events [4]. Simulation-based teaching provides nursing students valuable opportunities to think logically and make decisions regarding simulated clinical scenarios without risking patient safety. During post-scenario debriefings, students reflect on their experiences, discuss their strategies, and explore the emotions and behaviors influencing their decision-making processes. These discussions actively promote the development and application of critical thinking skills among students [1].

Self-efficacy refers to an individual’s belief in their ability to succeed in activities and achieve specific goals under particular conditions. According to Bandura [5], a high level of self-efficacy is developed through personal experiences and repeated successes. In nursing education, higher self-efficacy correlates positively with beneficial outcomes such as improvements in clinical practice, increased motivation in task selection and execution, and a readiness to view challenges as opportunities for growth. Self-efficacy is a crucial outcome that simulation-based training aims to promote among nursing students [6].

Previous studies have consistently reported positive effects of simulation-based training on nursing students’ problem-solving skills and self-efficacy, whereas the evidence regarding critical thinking skills is still contradictory. In Vietnam, several nursing schools have adopted simulation-based training, mainly for preclinical education. However, there are limited published studies examining the effect of simulation-based training in nursing education in Vietnam. Therefore, this study was conducted to provide additional evidence about this teaching method, aiming to inform improvements and improve the quality of nursing education in Vietnam.

### Objectives

This study aimed to compare students’ problem-solving skills, critical thinking skills, and self-efficacy before and after participating in a simulation-based training course on adult nursing.

## Methods

### Ethics statement

The study was granted approval by the Ethics Committee in Biomedical Research, Da Nang University of Medical Technology and Pharmacy, Vietnam (approval number: 646/BB-HĐĐĐ on February 20, 2021). Informed consent was obtained from the participants.

### Study design

This was a quasi-experimental study with a single-group pretest and posttest design. It is described according to the Transparent Reporting of Evaluations with Nonrandomized Design (TREND) statement, which is available at: https://www.cdc.gov/trendstatement/index.html.

### Setting

Data were obtained using a census sampling method at Da Nang University of Medical Technology and Pharmacy, one of the largest medical universities in Central Vietnam, from May to July 2022. This study targeted second-year full-time nursing students. Before practicing in the clinical setting, these students were required to finish the preclinical courses in medical and surgical nursing care, which are taught using a simulation-based training method. On the day before the preclinical course started, all the students attended an orientation hour, in which they were instructed on how to be involved and learn in simulation classes. At this time, they were invited to join the study. After providing informed consent, they were given the questionnaire.

### Participants

This study included 173 second-year nursing students who were required to complete the adult nursing simulation-based training course. Those who did not finish 8 sections of the course were excluded from the study.

### Interventions

All participants were divided into 8 groups (20–22 students in each group). An identical simulation-based training course on adult nursing was given to each group, consisting of 8 sections in the simulation rooms (2 sections per week and lasting for 4 weeks). Each section took place within 200 minutes (Supplement 1). The course used moderate-fidelity simulated teaching approaches (using moderately realistic simulation models), based on the World Health Organization guidelines [7]. The course was taught by simulation-trained instructors using the same scenarios for different groups. All scenarios were standardized by the training council of the department. Data were collected before and after the course was completed.

### Outcomes

The outcomes of this study were nursing students’ problem-solving skills, critical thinking skills, and self-efficacy.

### Measurement

Data were gathered using a 4-part questionnaire that included the participants’ characteristics, the Problem-Solving Inventory (PSI) under a RightsLink License [3], the Critical Thinking Skills Questionnaire (CTSQ) [4], and the General Self-Efficacy Scale (GSES) [8].

The PSI was used to measure nursing students’ problem-solving skills, which comprise 3 components: Problem-solving confidence (11 items), approach-avoidance style (16 items), and personal control (5 items). Problem-solving confidence is defined as confidence in one’s ability to solve difficulties and self-assurance when confronted with a variety of problem-solving activities. The approach-avoidance style is characterized as a general tendency to approach or avoid specific problem-solving tasks. Furthermore, confronting difficulties is associated with lower scores on this aspect, whereas avoiding problems is related to better scores. The third component of PSI, personal control, is defined as the conviction that one can manage one’s emotions and behaviors to solve problems. This component appears to be a representation of both emotional hyperactivity and behavioral control. The PSI consists of a total of 32 items designed on a 6-point Likert scale from 1 (strongly disagree) to 6 (strongly agree). The lowest possible score is 32 and the highest score is 192. A higher overall score indicates a lower level of problem-solving skills. The original scale had a Cronbach’s α reliability coefficient of 0.85 [3].

The CTSQ is a tool for assessing critical thinking skills by investigating activities that might be undertaken when thinking critically about a certain situation. The questionnaire consists of 34 items rated on a 5-point Likert scale, ranging from 1 (never) to 5 (always), indicating the frequency of these activities. The critical thinking skills score is calculated using the total points from all 34 items, with a higher total score reflecting better critical thinking skills. This tool was developed by Vietnamese authors using a unified definition of critical thinking skills from Delphi experts and Peter Honey’s critical thinking skills measurement toolkit. The tool showed a Cronbach’s α reliability coefficient of 0.87 [4].

The GSES is a 10-item assessment tool that measures an individual’s confidence in their abilities. Each item has 4 possible options, which are scored as follows: (1) completely incorrect, (2) almost incorrect, (3) somewhat correct, and (4) completely correct. Self-efficacy scores are computed by summing the responses to all 10 items. A higher score suggests that the individual is more self-confident. The Cronbach’s α of this tool has been reported to range between 0.76 and 0.90 [8].

After receiving permission from the authors, the PSI and GSES were translated into Vietnamese using the back translation technique [9]. Subsequently, the Vietnamese version of the PSI, the CTSQ, and the GSES were evaluated by 5 nursing educators with PhD (Doctor of Philosophy) or master’s degrees, yielding a content validity index of 1. A pilot study with 30 students was conducted to examine reliability, which resulted in Cronbach’s α values of 0.85, 0.94, and 0.90, respectively. Raw response data from participants were available at Dataset 1.

### Bias

To avoid selection bias, this study was conducted on the entire relevant population. Moreover, 3 questionnaires (PSI, CTSQ, and GSES) were verified for validity and reliability to minimize measurement bias.

### Study size

The sample size for this study was estimated using G*Power ver. 3.1.9.4 (Heinrich-Heine-Universität Düsseldorf; http://www.gpower.hhu.de/). The dependent t-test used a 2-tailed alpha of 0.05, with an effect size of 0.3, an α error probability of 0.05, and a power of 0.95. The result revealed that a sample size of 147 students was required. However, the researchers included all relevant students to boost the study’s generalizability. The sample had a total of 173 nursing students.

### Blinding (masking)

The participants were not blinded.

### Unit of analysis

The group served as an analysis unit.

### Statistical methods

Descriptive statistics were employed to describe all variables in this study. After confirming a normal distribution, the paired-sample t-test was conducted to compare the scores of problem-solving skills, critical thinking skills, and self-efficacy of participants before and after the intervention, with a significance threshold of P<0.05. All statistical analyses were carried out using IBM SPSS ver. 20.0 (IBM Corp.).

## Results

### Participants

All the participants completed the questionnaire. As shown in Table 1, the age of the participants ranged from 19 to 22 years, with a mean of 19.98±0.51 years. The majority of them were female (92.5%). At baseline, there was no significant difference between women and men in terms of problem-solving skills, critical thinking skills, and self-efficacy (Supplement 2).

### Main results

**Fig. 1.**
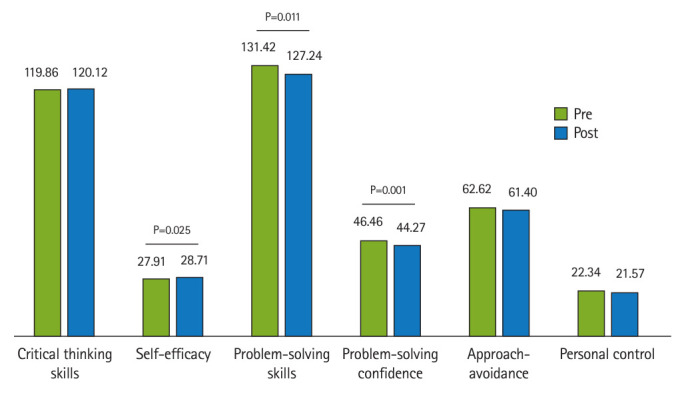
Differences in the participants’ problem-solving skills, critical thinking skills, and self-efficacy before and after the intervention.

Fig. 1 shows the changes in scores for problem-solving skills, critical thinking skills, and self-efficacy from the pretest to the posttest. After attending the simulation-based training course, the average problem-solving score (127.24±12.11) was lower than before (131.42±16.95), with a statistically significant difference (t_172_=2.55, P=0.011). Among the 3 components of problem-solving skills, there was a significant difference in problem-solving confidence after the simulation-based training compared to the baseline (t_172_=3.33, P=0.001).

In addition, the mean score of critical thinking skills increased from the pretest to the posttest; however, this change was not statistically significant (P=0.854). Furthermore, there was a significant increase in self-efficacy scores after taking the simulation-based training course compared to the baseline (t_172_=-2.26, P=0.025).

The differences in the participants’ problem-solving skills, critical thinking skills, and self-efficacy before and after the intervention are shown in Supplement 3.

## Discussion

### Key results

There were statistically significant improvements in the participants’ average scores for problem-solving skills and self-efficacy after taking the simulation-based training course. However, no significant change was observed in the scores for critical thinking skills.

### Interpretation

The average score for problem-solving skills after taking the simulation-based training course was significantly lower than the baseline, suggesting an improvement in participants’ problem-solving skills. Specifically, among the 3 components of problem-solving skills, a significant difference was only observed in problem-solving confidence. In our study, students engaged in diverse clinical scenarios during each session, covering various patient needs and care techniques. These scenarios were designed to vary in complexity, offering students opportunities to tackle challenges of differing difficulty levels. The simulation sessions, which involved pre- and post-scenario discussions, played a crucial role in developing students’ problem-solving skills by increasing their confidence in dealing with clinical issues.

In this study, another outcome measured was critical thinking skills. However, the results showed no significant difference in students’ critical thinking skills after completing the course compared to the baseline. This finding may be attributed to the relatively short duration of the course, which consisted of only 8 sessions conducted during a 4-week period. This situation could contribute to student fatigue, potentially hindering their ability to absorb information, practice, and develop skills, particularly critical thinking.

Self-efficacy is a targeted outcome that is commonly measured to examine the effect of simulation training on nursing students. In this study, we observed an increase in the average score for students’ self-efficacy after exposure to the simulation course, indicating an improvement. During simulation-based classes, students had opportunities to practice nursing skills repeatedly across diverse scenarios without putting patients at risk. In addition, engaging in solving given situations and group interactions allowed students to gain valuable experience, thereby increasing their confidence. Notably, students received immediate and constructive feedback from instructors and peers during debriefing sessions following scenario execution.

### Comparison with previous studies

The results of this study demonstrated a significant improvement in the problem-solving skills of participants following the course. This finding aligns with previous studies that have highlighted the success of simulation-based education in enhancing problem-solving skills among nursing students [2,10]. A study in Turkey concluded that using high-fidelity simulation improved nursing students’ problem-solving skills [2]. Similarly, a study involving Korean nursing students highlighted significant improvements in problem-solving abilities following a simulation-based training program focusing on integrated nursing practice [10].

Moreover, this study found an improvement in students’ self-efficacy after completing the simulation course. Our finding is consistent with some previous studies that have documented positive effects of simulation-based training on self-efficacy among nursing students [2,6,11].

In this study, there was no statistically significant change in students’ critical thinking skills after completing the simulation-based training course on adult nursing. This result is consistent with the findings from a meta-analysis including 38 studies [12]. However, contrasting results were reported in some previous studies around the world. A study in Malaysia found that simulation-based training using high-fidelity patient simulation improved critical thinking skills among nursing students [13]. The positive effect of simulation-based education on students’ critical thinking skills was also documented in other review studies [1,14]. The disparity between our findings and others may be attributed to the level of simulation fidelity. Our study utilized moderate-fidelity simulation teaching with 8 sessions conducted over a short period. It is possible that this duration was insufficient to observe changes in students’ critical thinking skills compared to the more intensive and immersive high-fidelity simulation teaching approach applied in other studies [1,13,14].

### Limitations

Using a single-group pre-and post-test design without a control group and randomization poses challenges in controlling for potential confounding variables or establishing causality. In addition, self-assessment tools have their limitations as they primarily measure how individuals perceive their abilities, but they cannot eliminate biases such as the Dunning-Kruger effect, nor can they accurately gauge the alignment between participants’ self-perception and their actual skills. Moreover, the short duration of the simulation-based training course is another limitation of our study.

### Generalizability

Since this study utilized a census sampling method with the largest possible sample size, its findings can reasonably be generalized to nursing education in Vietnam. However, it is important to acknowledge potential confounding factors related to simulations that may influence the outcomes. These factors should be carefully considered when interpreting and applying the study’s findings in a broader context.

### Suggestions

It is recommended that nursing education schools should consider adopting simulation-based training methods, particularly high-fidelity simulations, to effectively develop and improve students’ essential skills. Furthermore, future intervention studies should incorporate randomized control groups and extend over a longer period to comprehensively measure additional outcomes and assess the effectiveness of the simulation-based training approach more rigorously.

### Conclusion

Simulation-based training can improve the problem-solving skills and self-efficacy of nursing students. This teaching approach should be utilized in nursing education programs in Vietnam.

## Figures and Tables

**Figure f2-jeehp-21-24:**
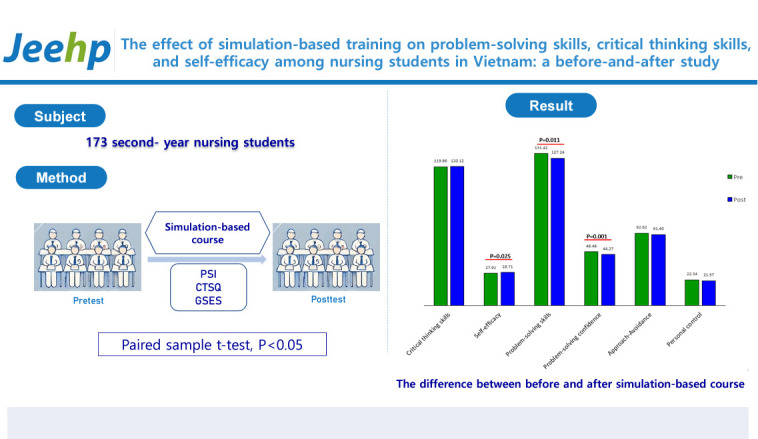


**Table 1.** Characteristics of the participants (n=173)

Characteristic	Value
Age (yr)	19.98±0.51
Gender	
Women	160 (92.5)
Men	13 (7.5)

Values are presented as mean±standard deviation or number (%).

## Footnotes

**Data availability**
Data files are available from Harvard Dataverse: https://doi.org/10.7910/DVN/XN49KQ Dataset 1. Raw responses data from participants.

## Supplementary materials

Supplementary files are available from Harvard Dataverse: https://doi.org/10.7910/DVN/XN49KQ

Supplement 1. Educational objectives and contents.

Supplement 2. The differences in problem-solving skills, critical thinking skills, and self-efficacy between male and female students at the baseline.

Supplement 3. The difference in the participants’ problem-solving skills, critical thinking skills, and self-efficacy before and after the intervention.

Supplement 4. Audio recording of the abstract.
