# Supplementary material for: The effect of simulation-based training on problem-solving skills, critical thinking skills, and self-efficacy among nursing students in Vietnam: a before-and-after study
Source: J Educ Eval Health Prof. 2024 Sep 23;21:24. doi: 10.3352/jeehp.2024.21.24 (PMC11480641; doi:10.3352/jeehp.2024.21.24)
Supplement: Supplementary file 2 — Supplement 1. Educational objectives and contents. [file jeehp-21-24-suppl1.docx]

**Supplement 1.** Educational objectives and contents

| Week | Sessions | Content | Time (min) |
| --- | --- | --- | --- |
| 1 | 1 | Nursing care for adult patients with cardiovascular internal medicine conditions | 200 |
|  | 2 | Nursing care for adult patients with cardiovascular surgical conditions | 150 |
| 2 | 3 | Nursing care for adult patients with respiratory internal medicine conditions | 200 |
|  | 4 | Nursing care for adult patients with respiratory surgical conditions | 150 |
| 3 | 5 | Nursing care for adult patients with gastrointestinal internal medicine conditions | 200 |
|  | 6 | Nursing care for adult patients with gastrointestinal surgical conditions | 200 |
| 4 | 7 | Nursing care for adult patients with urological internal medicine conditions | 200 |
|  | 8 | Nursing care for adult patients with urological surgical conditions | 200 |

The main goals of each section were to provide care for patients with internal or surgical diseases with 3 main objectives: (1) assessing the health status of adults with related diseases, (2) implementing evidence-based nursing procedures, and (3) cultivating urgency and autonomy in handling clinical situations.
