# Supplementary material for: The effect of simulation-based training on problem-solving skills, critical thinking skills, and self-efficacy among nursing students in Vietnam: a before-and-after study
Source: J Educ Eval Health Prof. 2024 Sep 23;21:24. doi: 10.3352/jeehp.2024.21.24 (PMC11480641; doi:10.3352/jeehp.2024.21.24)
Supplement: Supplementary file 3 — Supplement 2. The differences in problem-solving skills, critical thinking skills, and self-efficacy between male and female students at the baseline. [file jeehp-21-24-suppl2.docx]

**Supplement 2.** The differences in problem-solving skills, critical thinking skills, and self-efficacy between male and female students at the baseline

| Variable | Mean±SD | | t-value | df | P-value |
| --- | --- | --- | --- | --- | --- |
|  | Male | Female |  |  |  |
| Problem-solving skills | 135.67±15.87 | 131.10±17.03 | 0.90 | 171 | 0.37 |
| Problem-solving confidence | 48.08±4.78 | 46.34±6.79 | 0.86 | 171 | 0.38 |
| Approach-avoidance style | 65.67±10.26 | 62.39±11.24 | 0.17 | 171 | 0.33 |
| Personal control | 21.92±2.72 | 22.37±3.24 | 0.92 | 171 | 0.65 |
| Critical thinking skills | 121.08±12.64 | 119.76±13.66 | 0.32 | 171 | 0.746 |
| Self-efficacy | 28.83±6.60 | 27.84±5.16 | 0.63 | 171 | 0.529 |

SD, standard deviation; df, degrees of freedom.
