# Supplementary material for: The effect of simulation-based training on problem-solving skills, critical thinking skills, and self-efficacy among nursing students in Vietnam: a before-and-after study
Source: J Educ Eval Health Prof. 2024 Sep 23;21:24. doi: 10.3352/jeehp.2024.21.24 (PMC11480641; doi:10.3352/jeehp.2024.21.24)
Supplement: Supplementary file 4 — Supplement 3. The difference in the participants’ problem-solving skills, critical thinking skills, and self-efficacy before and after the intervention. [file jeehp-21-24-suppl3.docx]

**Supplement 3.** The difference in the participants’ problem-solving skills, critical thinking skills, and self-efficacy before and after the intervention

| Variable | Mean±SD | | t-value | df | P-value |
| --- | --- | --- | --- | --- | --- |
|  | Pre-test | Post-test |  |  |  |
| Problem-solving skills | 131.42±16.95 | 127.24±12.11 | 2.55 | 172 | **0.011** |
| Problem-solving confidence | 46.46±6.67 | 44.27±5.61 | 3.33 | 172 | **0.001** |
| Approach-avoidance style | 62.62±11.81 | 61.40±7.23 | 1.19 | 172 | 0.236 |
| Personal control | 22.34±3.20 | 21.57±4.42 | 1.85 | 172 | 0.066 |
| Critical thinking skills | 119.86±13.56 | 120.12±13.92 | -0.18 | 172 | 0.854 |
| Self-efficacy | 27.91±5.26 | 28.71±3.81 | -2.26 | 172 | **0.025** |

Statistically significant results are marked in bold.

SD, standard deviation; df, degrees of freedom.
